# Automation of Dead End Filtration: An Enabler for Continuous Processing of Biotherapeutics

**DOI:** 10.3389/fbioe.2020.00758

**Published:** 2020-07-03

**Authors:** Garima Thakur, Vishwanath Hebbi, Subhash Parida, Anurag S. Rathore

**Affiliations:** Department of Chemical Engineering, Indian Institute of Technology Delhi, New Delhi, India

**Keywords:** Dead end filtration, continuous bioprocessing, depth filtration, turbidity breakthrough, pressure breakthrough

## Abstract

Dead end filtration is a critical unit operation that is used for primary and secondary clarification during manufacturing of both microbial and mammalian cell based biotherapeutics. Dead end filtration is conventionally done in batch mode and requires filter pre-sizing using extensive scouting studies, along with filter over-sizing before deployment to handle potential variability. However, continuous manufacturing processes require consistent use of dead-end filtration over weeks or months, with potential unpredictable variations in feed stream attributes, which is a challenge currently facing the industry. In this work, a dead-end filtration skid is designed for continuous depth filtration, incorporating multiple small-sized filters along with turbidity, and pressure sensors with immediate switching to a fresh filter whenever turbidity or pressure breakthrough above a pre-determined cut-off is detected in real time. The skid has been successfully tested for manufacturing of granulocyte colony stimulating factor from *Escherichia coli*, human serum albumin from *Pichia pastoris*, and a monoclonal antibody therapeutic from CHO cells. The proposed skid can be directly applied for any dead-end filtration application with minimal prior scouting studies or sizing calculations for scale-up. It is a useful solution for continuous processing trains where the nature of the feed, such as its turbidity or host cell proteins content, may change over long continuous campaigns, rendering previous sizing calculations inaccurate. The skid also allows significant cost savings by eliminating the sizing safety factor of 1.5–2x which is generally added before filter deployment at manufacturing scale.

## Introduction

The success of biologic molecules, in particular monoclonal antibodies (mAbs), in treating various diseases has led to an increased demand and need for scale-up technologies (Ashton, 2001; Anicetti, 2009; Roger, 2010; Xu and Zhang, 2014). Increasing demand has driven significant improvements in upstream process, with current commercial titers for mAbs as high as 10 g/L (Birch and Biologics, 2005; Kelley, 2009; Pollock et al., 2013; Tran et al., 2014; Grilo et al., 2017) compared to the older processes that would have titers of <1 g/L. The titers of microbial processes have also risen up to 5–6 g/L for both *Escherichia coli* (Slouka et al., 2018; Wilson et al., 2019) and *Pichia pastoris* based platforms (Kjeldsen, 2000; Kjeldsen et al., 2002; Xie et al., 2008). High cell density is now commonly achieved in both mammalian and microbial processes, and therefore primary recovery of cells can be a significant challenge (Wurm, 2004; Aldor et al., 2005). At manufacturing scale, mammalian, bacterial, and yeast-based harvest broths are all traditionally clarified by using depth filters. Introduction of depth filters in the downstream processing train has shown to increase the capacity of the subsequent sterilizing grade filters which are typically used to protect chromatography columns and viral filters from clogging (Kandula et al., 2009; Pegel et al., 2011). Depth filters are used not only to remove insoluble widely distributed particles in the train, but can also be exploited for removing soluble impurities such as host cell proteins (HCP), host cell DNA (HCDNA), and charged particles (Yigzaw et al., 2006; Khanal et al., 2018).

Despite the ubiquity of depth filtration, accurate sizing of the filters remains a challenge. At manufacturing scale, sizing is very important for economical utilization of filter modules, which are one of the most expensive consumables in the downstream process after chromatography resins, and can contribute up to 50% of the consumable costs for downstream clarification (Felo et al., 2013). Filter capacity and sizing studies are affected by a large number of parameters, including the type of cell line, culture conditions, aggregate levels, particle size distribution, centrifugation parameters, and lot-to-lot filter variability (Yavorsky et al., 2003; Goldrick et al., 2017). It is difficult to accurately account for potential changes in parameters such as cell viability which may lead to higher amounts of HCP and HCDNA and lead to increased turbidity of the process stream (Boerlage, 2001), thereby affecting the sizing requirements (Boerlage, 2001; Pham, 2010; Krupp et al., 2017). Over-sizing of depth filters can significantly impact the economics of the process, while under-sizing can lead to process deviations such as turbidity breakthrough, clogging of subsequent sterile filters, or fouling of chromatographic columns and ultrafiltration filters, significantly hampering product quality, and process productivity (Wakeman, 2007; Kandula et al., 2009; Lutz, 2009; Popova et al., 2016).

In industry, depth filtration sizing is currently performed by using representative samples of the process stream at small scale. Filters are usually operated under constant flow rate conditions so that volumetric flow rates remain constant over time, allowing consistent operation of subsequent downstream steps (Liu et al., 2010). For constant flow operation, the filter capacity is defined by the cumulative volume of filtrate until a maximum pressure rating or turbidity breakthrough is reached, whereas for constant pressure, the filter capacity is defined as the total volume of feed processed until the flow at that pressure becomes lower than a certain minimum cut-off. Once the process development is completed at small scale, linear scale-up is possible along with an extra safety factor of 1.5 to 2 times the linearly scaled-up filter area, as a benchmark based on manufacturing experience (Lutz, 2009; Agarwal et al., 2016). A larger safety factor increases the process resilience to deviations and reduces the risk of process material exceeding the filter capacity. However, large safety factors also significantly increase process costs due to the high cost of filters, as well as increase product loss, holdup volumes, and process preparation downtime.

Current depth filtration approaches also fall short when it comes to continuous processing trains (Jungbauer, 2013; Croughan et al., 2015; Konstantinov and Cooney, 2015). The shift from batch to continuous has been facilitated by a combination of enabling technologies such as multi-column chromatography setups, and process analytical technology (PAT) tools for real time monitoring of critical quality attributes and process parameters in accordance with the US FDA’s guidance on PAT (FDA, 2004). However, adapting dead-end depth and sterile filtration for continuous operation has been neglected thus far. There have been some attempts to implement PAT approaches in dead-end filtration by incorporating in-line monitoring sensors to trigger alarms in case process limits are exceeded. For example, in-line pressure sensors have been used to monitor differential pressure across dead-end filters in real time, plot the data on monitoring charts, and trigger alarms in case of pressure build-up due to filter clogging or other issues such as kinks in tubing (Bink and Furey, 2010). Another approach utilized in-line absorbance measurements at 410 nm to measure HCP breakthrough through depth filters, as HCP complexes give detection signals at this wavelength but the target proteins do not (Rathore et al., 2010).

However, a complete solution for continuous operation of dead-end filtration has not yet been developed. The solution must incorporate enabling technology to allow process flow streams to pass through the filters without interruption over weeks or months of continuous operation (Steinebach et al., 2017). The setup should also be equipped with relevant sensors to monitor turbidity or pressure in the feed and filtrate streams and ensure that filters are replaced, either automatically or manually, when they become clogged. This should be done without affecting the filtrate quality at any point in the continuous operation, as any turbidity breakthrough or filter damage can lead to clogging of the subsequent unit operations like chromatography columns. Finally, the setup should be flexible and robust enough to handle unexpected deviations or shifts in the composition of the feed stream, such as increased turbidity or titer due to changing cell culture conditions in the upstream reactor. The filter should not rely on extensive sizing calculations, which are unlikely to be accurate in the case of months of continuous operation instead of well-characterized batches. In this paper, we propose such a filtration skid setup with three small-size filters (each with less than 10% of the required large-scale size) and integrated in-line pressure and turbidity sensors. The process flow stream is directed through one filter at constant flowrate until a pressure or turbidity breakthrough set-point is reached, at which time a valve instantly switches and directs the stream through a waiting, pre-equilibrated filter. The process can therefore run uninterrupted without any downtime or disruption in flow. Due to this continuous cycling of filters, smaller sized filters can be used and significant savings can be made in overall filter area as some filters will likely remain unused. We have demonstrated the application of the skid in various downstream steps, including harvest clarification and sterile filtration for *E. coli, P. pastoris*, and Chinese hamster ovary (CHO) cell expressed products.

## Materials and Methods

### Materials

#### Electrical Components

Micrologix 5000 from Allen Bradley with analog extension modules; 24V power supply unit, extension module, multiplex, and necessary electrical components and wiring were procured from a local vendor.

#### Filters

B1HC, D0HC, B0HC, and X0HC were procured from Millipore Corporation; 90ZA was procured from 3M Company; HP PDD1, HP PDH4, and Sterile Acrodisc Syringe filter 4652 were procured from Pall Life Sciences.

### Feed Materials

#### rGCSF Refolding Output

The rGCSF was produced in the form of inclusion bodies (IBs). IBs were solubilized and refolded using dilution method (Hebbi et al., 2019). After the peak point was reached (8–10 h), the refolding reaction was quenched using glacial acetic acid at pH 4. In this process, many HCP were precipitated, which are generally removed using depth filtration.

#### CHO Cell Culture Harvest

Monoclonal antibody cell culture harvest was obtained from a major biopharmaceutical company. Two types of cell clarification studies were conducted, first using direct cell culture harvest for filtration and the second using post-centrifugation centrate for filtration.

#### Pichia Broth

*Pichia pastoris* broth for HSA production was directly taken from bioreactor with 20.3% wet weight. Another study was conducted using centrate from the centrifugation of the broth as it was noticed that the centrate is generally unstable and becomes turbid, which demands depth filtration post-centrifugation before processing the process feed by capture chromatography.

#### rGCSF Drug Product

Sterile filtration was conducted for rGCSF purified drug product after inclusion body solubilization, protein refolding, and polishing chromatography steps.

#### mAb Drug Product

Sterile filtration was conducted for mAb drug product after centrifugation, Protein A capture chromatography, viral inactivation, and polishing chromatography steps.

### Method for Pressure and Turbidity Excursion Studies

When designing a clarification scheme, the properties of the particulate matter in the process stream play a significant role in defining the outcome. Particle size, density, and nature of the process stream vary as the stream moves from upstream to the final product. For example, for clarified mammalian cell culture broth, the number of cells, cell viability, HCP levels, and HCDNA levels are key feed attributes. For high cell density fermentations like *P. pastoris*, solid content in terms of wet weight percentage is critical. For already clarified feed streams like chromatography elutes or quenched refolding output, optical density at 600 nm or turbidity of the feed stream can provide the required input. In many cases, tools for measuring particle size distribution are used to get insight into the mechanism of clogging, which can be very useful for selecting the optimal filter for improved performance. Once a set of depth filters have been selected, screening can be started to determine the final filter choice for use in the proposed continuous skid.

Properties of the different feed streams in our study are listed in Table 1. In general, process streams with high cell densities are highly unstable with particle sizes in micron ranges and have to be clarified immediately after harvest. For streams after centrifugation, the particle size reduces to less than 100 nm with nephelometric turbidity units (NTU) of 200–400. The refolding output of GCSF has particle size of 50–100 nm and NTU of 1600. The GCSF drug product, on the other hand, is highly clarified as it is the final formulated product and needs to be filtered through sterile filtration before filling and packaging. Filter screening can also be performed for primary and secondary filters in series. In our trials, 90ZA, and B0HC filters were screened as secondary depth filters for harvest material after the primary depth filter 30S, which is a commonly used setup in mAb processing. An alternative approach is that to perform centrifugation followed by a single depth filtration. Depth filters X0HC, B1HC, and B0HC were used as depth filters for filtering the centrate of mAb harvest.

**TABLE 1:** Properties of different feed streams used in this study.

**Feed stream**	**Physicochemical properties of particulate matter**
*Pichia pastoris* cell broth for HSA production	NTU 3500, mean particle size 4–6 μm w/w
*Pichia pastoris* cell broth centrate	NTU 300, mean particle size < 100 nm, unstable, becomes hazy in 12–15 h
CHO cell culture harvest	NTU 1785, cell viability 85%
CHO cell culture centrate	NTU 200–400, unstable, becomes hazy in 12–15 h
Refolding output of GCSF (quenched)	NTU 1600, white precipitate due to HCP while quenching from 9.5–4 using acetic acid, 50–100 nm size
Drug product GCSF	Clear solution
Drug product mAb	Clear solution

Process samples and their corresponding filters were selected and screened for pressure breakthrough (for secondary depth filters and sterile filters) and turbidity breakthrough (for primary depth filters). Constant-flux turbidity excursion trials were conducted to quantify the cut-off NTU value where turbidity breakthrough starts. Primary depth filters are often vulnerable to turbidity breakthrough before pressure breakthrough. Thus, their capacities are measured based on NTU cut-off rather than pressure cut-off, as high NTU in the post-filter stream can clog subsequent filters or unit operations. In our demonstrations, a cut-off of 1000 NTU was selected for turbidity breakthrough. Similarly, pressure excursion studies were performed for secondary depth filters and sterile filters and a pressure cut-off of 1.5 bar was selected due to operational limitations of the filtration setup and tubing. The cut-offs can be adjusted on a case-by-case basis.

For the excursion studies, filters were washed with WFI followed by the respective buffer at 200 LMH. The washed filter was connected to the skid after the solenoid valve. The process stream was pumped through the skid using a peristaltic pump with flow rate of 200 LMH in the case of depth filtration and 300 LMH in the case of sterile filtration. The pressure and turbidity values were recorded in real time. High performance liquid chromatography (HPLC) methods for GCSF (Hebbi et al., 2019), HSA (Chopda et al., 2017), and mAb (Kateja et al., 2016) were used for analyzing the concentrations of product in the feed and filtrate.

### Set-Up and Demonstration of the Continuous Skid

**FIGURE 1:**
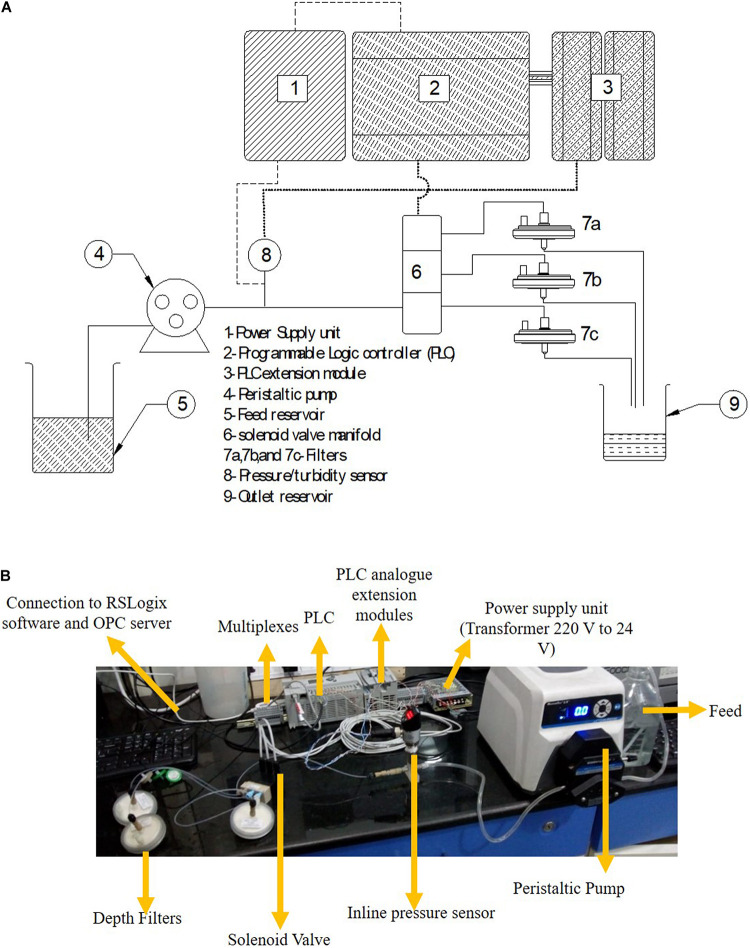
**(A)** Schematic and **(B)** Image of the experimental set up.

The schematic of the skid is shown in Figure 1A and the actual set up is shown in Figure 1B. A MicroLogix 5000 programmable logic controller (PLC) from Allen Bradley was operated by using Micrologix software. The software was layered with an OPC server (MatrikonOPC Explorer) for continuous data collection and logging. The power supply unit was connected to the PLC, pressure transmitter, and turbidity sensor. The signal from the PLC was transferred to the solenoid valve which allowed for switching of the flow stream between filters. The PLC acquired the data from the pressure transmitter and turbidity probes. The turbidity and pressure values were compared every second in real time to the cut-off value selected for the particular sample and corresponding filter. Once the current value exceeded the cut-off value, the PLC passed a signal to the solenoid valve to trigger switching of the flow stream through a fresh filter.

## Results and Discussion

### Turbidity and Pressure Excursion Studies

Constant-flux turbidity and pressure excursion trials were carried out for a range of feed materials for both depth and sterile filtration. Multiple process streams from CHO cell, *P. pastoris* and *E. coli* expression platforms were used to demonstrate turbidity or pressure breakthrough of depth and/or sterile filters with appropriate pore sizes. Table 2 shows the comprehensive list of turbidity and pressure cut-offs and the corresponding filter capacities for the trials.

**TABLE 2:** Pressure or turbidity breakthroughs for different filters using different feeds.

**System**	**Filter ID**	**Type**	**Filter specification**	**Feed material**	**Selected cut-off**	**Filter capacity (L/m^2^)**	**Yield (%)**
mAb – *CHO cell*	B1HC	Secondary	0.1–0.7 μm, 23 cm^2^	Centrate of mAb CHO cell culture broth	1.5 bar	266.7 ± 6	95.23
	90ZA	Secondary	0.1–0.5 μm, 23 cm^2^	mAb CHO cell culture harvest filtered through 30S (harvest→30S)	1.5 bar	166.7 ± 9	92.45
	D0HC	Primary	0.5–10 μm, 23 cm^2^	mAb CHO cell culture harvest	1000 NTU	130.1 ± 5	91.63
	B0HC	Secondary	0.1 μm, 23 cm^2^	mAb CHO cell culture harvest filtered through 30S (harvest→30S)	1.5 bar	97.2 ± 6	90.67
	X0HC	Secondary	0.1 μm, 23 cm^2^	mAb CHO cell culture centrate	1.5 bar	233.4 ± 19	96.45
	B0HC	Secondary	0.1 μm, 23 cm^2^	mAb CHO cell culture centrate	1.5 bar	114.0 ± 7	102.38
	Acrodisc 4652	Sterile	0.2 μm, 2.8 cm^2^	mAb formulated drug product	1.5 bar	68.2 ± 4	104.67
Human serum albumin (HSA) – *Pichia pastoris*	EK1P	Primary	0.2–4 μm, 26 cm^2^	HSA *Pichia pastoris* broth with 5% (wet weight basis) solids	1000 NTU	23.3 ± 0.9	90.78
	HP PDH4	Secondary	0.4–15 μm, 26 cm^2^	HSA *Pichia pastoris* broth centrate	1.5 bar	35.5 ± 10	91.89
GCSF – *E. coli*	HP PDD1	Secondary	0.1–0.85 μm, 26 cm^2^	GCSF quenched refolding output	1.5 bar	146.7 ± 28	103.56
	HP PDH4	Secondary	0.5–15 μm, 26 cm^2^	GCSF quenched refolding output	1.5 bar	360.5 ± 32	98.49
	Acrodisc 4652	Sterile	0.2 μm, 2.8 cm^2^	GCSF formulated drug product	1.5 bar	165.2 ± 14	99.67

**FIGURE 2:**
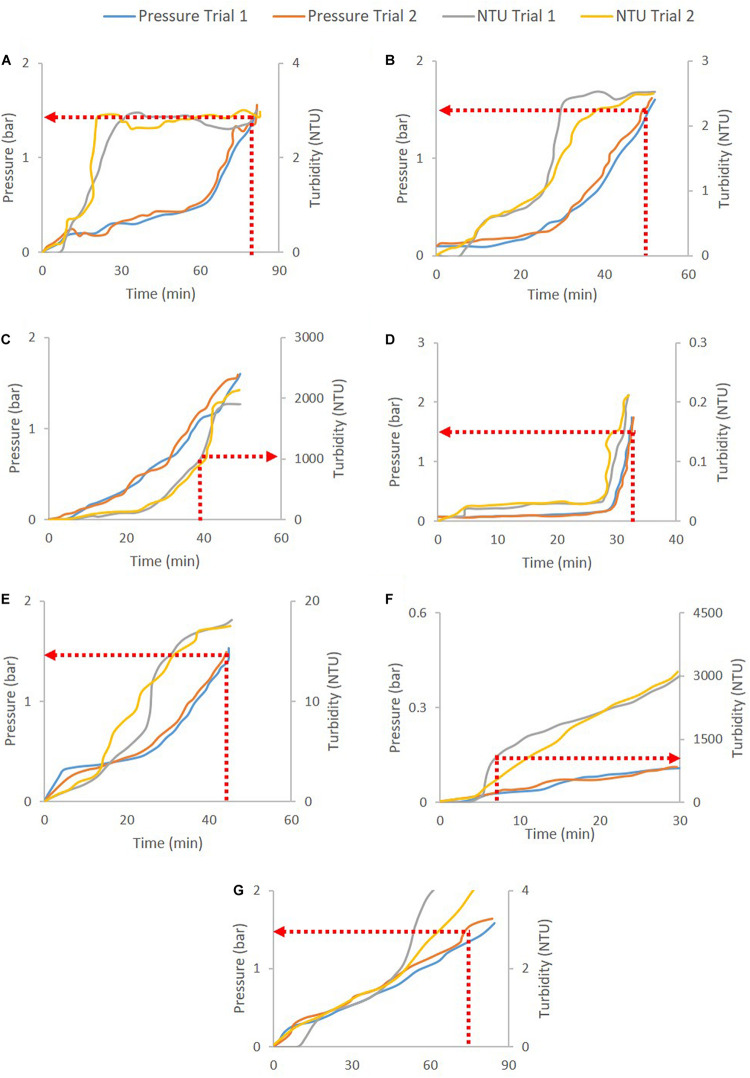
Depth filtration pressure and turbidity profiles for different cases: **(A)** B1HC centrate of mAb cell culture broth, **(B)** 90ZA Output of harvest filtered through 30S (harvest30S90ZA), **(C)** D0HC CHO cell culture harvest, **(D)** Acrodisc GCSF drug product, **(E)** HP PDD1 GCSF quenched refolding output, **(F)** HP PDH4 *Pichia pastoris* HSA broth with 5% solids, and **(G)** X0HC cell culture harvest centrate. The respective cut-offs are shown in dotted red lines and arrows. Pressure cut-off of 1.5 bar is used for secondary depth and sterile filters, while turbidity cut-off of 1000 NTU is used for primary depth filters.

Figure 2 shows the pressure and turbidity profiles from the filter studies. Figures 2C,F show the primary depth filters used for clarifying CHO cell culture harvest (D0HC) and *P. pastoris* broth with 5% solids (HP PDH4). In both cases, turbidity breakthrough can be seen to occur prior to pressure breakthrough. In Figures 2A,B,D–F, pressure cut-off is considered for operation. Duplicate experiments were conducted in each case. It can be seen from Figure 2 that the breakthrough profiles were similar across the duplicate pairs. The filter capacities corresponding to the cut-off points for the filters are listed in Table 2. The average and standard deviation of capacity at cut-off for the two trials was calculated in each case. The standard deviation was around 10% of the average capacity in most trials, suggesting that utilizing turbidity of pressure cut-offs for filter switching does not affect the reliability of the capacity studies carried out in the filter screening phase. The cut-off values were program into the PLC using ladder logic to continuously monitor the readings from the pressure and turbidity sensors, and trigger the solenoid valve to switch the flow to a fresh filter when the current value exceeded the program cut-off.

### Implementation of Skid in Continuous Process

**FIGURE 3:**
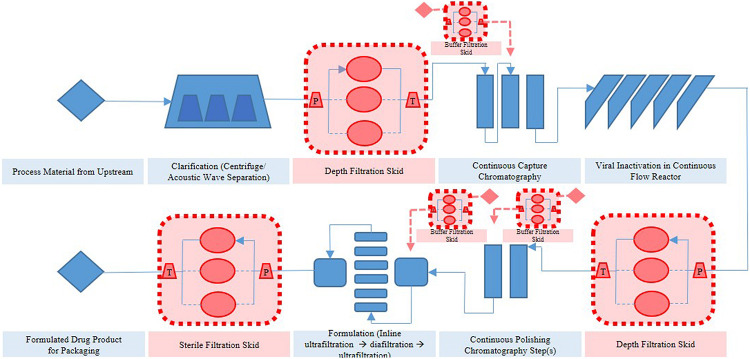
Applications of dead end filtration skid in continuous train.

**FIGURE 4:**
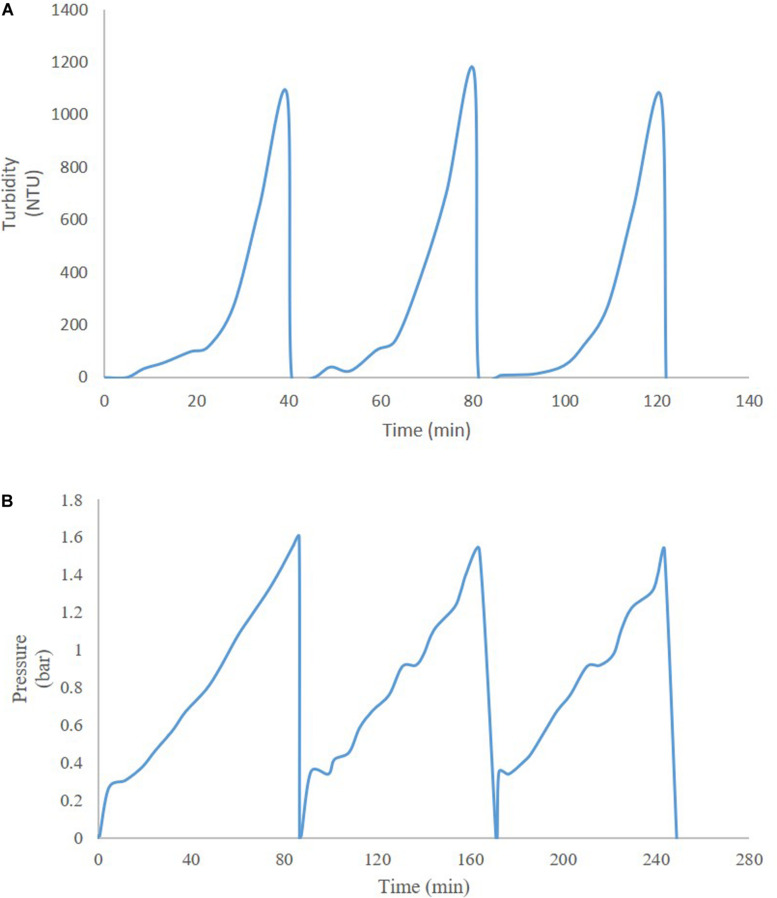
Profiles of continuous operation with **(A)** turbidity-based switching for HP PDH4 (Sample: HSA Pichia pastoris broth with 5% solids on wet weight basis), and **(B)** pressure-based switching for X0HC (Sample: mAb CHO cell culture centrate).

**FIGURE 5:**
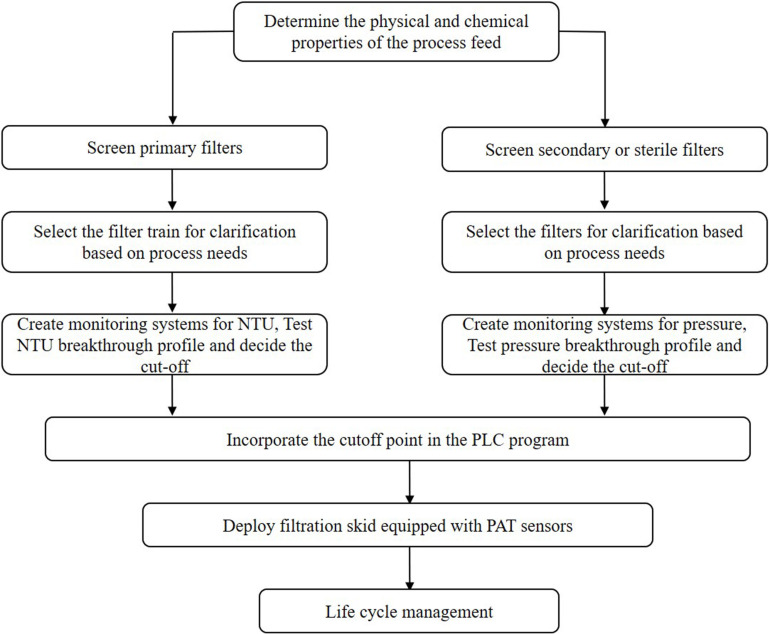
Process flow chart for implementation of continuous dead end filtration using the developed skid.

Thus far, several research groups have proposed filter sizing for continuous processing on the basis of campaign duration, along with a filter area safety factor of 1.5 to 2x (Lutz, 2009). Though this sizing and safety factor method has long been in place for batch processing, it is suboptimal in continuous processing, particularly during deviations in particle size, campaign duration, bioburden levels, or flowrate, all of which have high probability of occurring downstream of continuous perfusion cultures. The proposed skid is able to overcome these issues without prior knowledge or characterization of potential deviations, as it will simply increase the rate of filter switching if fouling increases, while ensuring consistent filtrate quality in the downstream steps due to real-time monitoring of pressure and turbidity breakthrough as the trigger for filter switching. Figure 3 shows a typical mAb continuous processing train and the multiple filtration steps where the proposed skid can be easily integrated. Figure 4 shows continuous switching profiles for both turbidity-based and pressure-based switching implementations of our proposed skid. The flowchart in Figure 5 illustrates the complete process for implementing the skid at any given point, starting from deciding the filter type based on physicochemical properties of the feed, followed by screening of filters, determination of the pressure or turbidity cut-off point, and program the skid.

### Economics of Batch Versus Continuous Dead-End Filtration

Filters typically are the second-most expensive consumable in downstream processing of mAbs, after Protein A chromatography resins (Kelley, 2007). When doing filter sizing, a safety factor 1.5–2x is considered for deciding the final area, increasing the filter cost. By using our proposed skid for filtration, significant savings in membrane area can be made as the filter area will be split into multiple filters out of which some may remain unused during the run. The installation of the skid and valve is a one-time capital cost which is likely to be offset by the subsequent savings in consumable costs over months or years of continuous operation. Furthermore, the same skid can easily be transferred between facilities or used for different molecules by simply changing the filters plugged into the setup. However, the added complexity of the skid and the need for GMP validation of the increased components and tubing lines lead to additional costs. Therefore, selecting the size of the filter to be used in the skid is a critical decision, and should be made at the sweet spot between cost savings from area reduction and cost increase due to additional skid complexity leading to increased labor and GMP validation costs.

**FIGURE 6:**
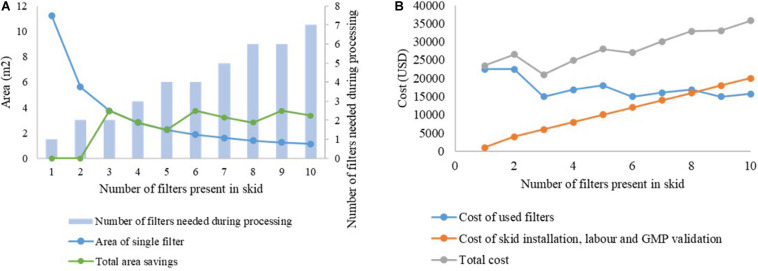
Cost analysis for selection of number of filters in the proposed skid. **(A)** analysis of area savings, **(B)** variation in overall costs with increasing number of filters in the skid.

This is illustrated in Figures 6A,B. The capacity of the filter B1HC for processing mAb CHO cell harvest centrate was found to be 266.7 L/m^2^, as shown in Table 2. In traditional batch processing, a safety factor of 1.5x would be applied to determine the final area of the depth filter to be installed. Therefore, to process 2,000 L of material in a batch facility would require 11.25 m^2^ of filter area. Using the proposed skid with filters of increasingly smaller size would lead to savings in membrane area up to the limiting value of 7.5 m^2^, corresponding to the area required as per the calculations of filter capacity with zero safety factor, as shown in Figure 6A. Assuming a fixed cost of USD 2,000 per m^2^ for the filter media, and a fixed cost of USD 1,000 for each new line in the skid (including installation, labor and GMP validation costs), the cost curve shown in Figure 6B is obtained. It can be seen that *n* = 3 is the optimum number of filters for this example. Similar analyses can be done on a case-by-case basis for any given application.

## Conclusion

Dead end filtration is a critical unit operation in manufacturing of biotherapeutics, and is used in both microbial and mammalian expression systems. Dead end filtration is challenging to implement in continuous processing due to the difficulty in maintaining a constant filtrate stream across the length of a campaign with a single filter. Adding large safety factors to filter sizes results in significant increase in cost of consumables. We propose a continuous dead end filtration skid consisting of multiple small filters and in-line pressure and turbidity sensors to monitor breakthrough in real-time and use pressure or turbidity cut-off values as a trigger to direct the feed stream to a fresh filter. The proposed skid allows easy handling of cell culture broth from continuous perfusion reactors that can have variable particle sizes, and eliminates downtime during filter change, water wash and buffer wash steps. It fills the gap between unit operations in the continuous train. It also provides a significant area reduction. The skid is versatile and can be applied for depth filtration, sterile filtration, buffer filtration, and other types of dead-end filtration across the manufacturing train using either pressure or turbidity breakthrough. Finally, the system is modular and can easily be applied to scaled-up systems. The proposed skid is a significant enabler for continuous processing.

## Data Availability Statement

The raw data supporting the conclusions of this article will be made available by the authors, without undue reservation.

## Author Contributions

GT and VH performed the experiments. SP contributed to program of the controllers. GT wrote the first draft. AR supervised the project, got the funding and reviewed and edited the final manuscript. All authors contributed to the article and approved the submitted version.

## Conflict of Interest

The authors declare that the research was conducted in the absence of any commercial or financial relationships that could be construed as a potential conflict of interest.
